# Alkaline ceramidase (*ClAC*) inhibition enhances heat stress response in *Cyrtorhinus lividipennis* (Reuter)

**DOI:** 10.3389/fphys.2023.1160846

**Published:** 2023-05-09

**Authors:** Min Chen, Xiao-Xiao Shi, Ni Wang, Chao Zhang, Zhe-Yi Shi, Wen-Wu Zhou, Zeng-Rong Zhu

**Affiliations:** ^1^ State Key Laboratory of Rice Biology and Breeding, Key Laboratory of Molecular Biology of Crop Pathogens and Insects, Ministry of Agriculture, Institute of Insect Sciences, Zhejiang University, Hangzhou, China; ^2^ Zhejiang Academy of Forestry, Hangzhou, China; ^3^ Hainan Research Institute, Zhejiang University, Sanya, China

**Keywords:** ceramidase, *Cyrtorhinus lividipennis*, heat stress, catalase, ceramide, nature enemy

## Abstract

Ceramidases (CDases) are vital sphingolipid enzymes involved in organismal growth and development. They have been reported as key mediators of thermal stress response. However, whether and how CDase responds to heat stress in insects remain unclear. Herein, we identified two CDase genes, *C. lividipennis* alkaline ceramidase (*ClAC*) and neutral ceramidase (*ClNC*), by searching the transcriptome and genome databases of the mirid bug, *Cyrtorhinus lividipennis*, an important natural predator of planthoppers. Quantitative PCR (qPCR) analysis showed that both *ClNC* and *ClAC* were highly expressed in nymphs than in adults. *ClAC* was especially highly expressed in the head, thorax, and legs, while *ClNC* was widely expressed in the tested organs. Only the *ClAC* transcription was significantly affected by heat stress. Knocking down *ClAC* increased the *C. lividipennis* nymph survival rate under heat stress. The transcriptome and lipidomics data showed that the RNA interference-mediated suppression of *ClAC* significantly upregulated the transcription level of *catalase* (*CAT*) and the content of long-chain base ceramides, including C16-, C18-, C24-, and C31- ceramides. In *C. lividipennis* nymphs, *ClAC* played an important role in heat stress response, and the upregulation of nymph survival rate might be caused by variation in the ceramide levels and transcriptional changes in *CDase* downstream genes. This study improves our understanding of the physiological functions of insect CDase under heat stress and provides valuable insights into the nature enemy application.

## 1 Introduction

Rice (*Oryza sativa*) is the most important food crop in the world with over half of the global population depending on this food resource (Project, 2005; Lou et al., 2014). The brown planthopper (BPH), *Nilaparvata lugens*, not only directly feeds on the rice plant but also transmits plant viruses (Liu et al., 2018). Since the Green Revolution, pesticide abuse increased the insect drug resistance and caused BPH outbreaks, which have seriously threatened rice production and affected human life (Bottrell and Schoenly, 2012). Thus, the biological control of BPH has increasing importance and is considered the sustainable strategy to avoid the shortcomings of chemical control (Lou et al., 2014). *Cyrtorhinus lividipennis* (Reuter), a hemipteran predator, is a dominant natural enemy of BPH. It feeds on BPH eggs and nymphs, thus effectively controlling the BPH population and playing an important role in BPH biological control (Matsumura et al., 2005; Sigsgaard, 2007; Preetha et al., 2010; Liu et al., 2018). However, with the acceleration of global warming, thermal stress is weakening the fitness and predatory capacity of *C. lividipennis* (Bai et al., 2022). Therefore, it is urgently required to investigate how *C. lividipennis* reacts to heat stress such as identifying the key resistance effectors to increase its survival rate under heat stress and improve its predation performance.

Sphingolipids play structural roles in cellular membranes and act as bioactive signaling molecules involved in multiple cell regulatory functions (Bartke and Hannun, 2009). Ceramides, located in the central part of sphingolipid metabolism, are the precursors of multiple complex sphingolipids (Kitatani et al., 2008; Gault et al., 2010; Young et al., 2013). Many research studies have demonstrated that sphingolipids participate in the regulation of thermal stress response. CDases are the most important metabolic enzymes of ceramides, hydrolyzing ceramides into free fatty acids and sphingosine (Mao and Obeid, 2008). CDases are divided into three subfamilies, including acid CDase (aCDase), neutral CDase (nCDase), and alkaline CDase (alCDase). nCDase have proved to be involved in the heat stress response of BPH (Shi et al., 2018). Serine palmitoyltransferase (*SPT*), localized in the first step of sphingolipid biosynthesis, is required for the accumulation of trehalose, a thermoprotectant in yeast cells under heat stress (Dickson et al., 1997; Jenkins et al., 1997; Dickson et al., 2006). Sphingosine-1-phosphate (S1P), a product of sphingosine (Sph) phosphorylation, enhanced the survival rate of *Arabidopsis* cell under heat stress by reducing programmed cell death (Alden et al., 2011). External addition of S1P could reduce the deleterious effect of heat stress on the development of bovine oocytes (Roth and Hansen, 2004). In mouse cells, ceramide and S1P activated the synthesis of heat shock proteins (HSPs) during heat shock response (Chang et al., 1995; Kozawa et al., 1999). The aforementioned data indicate the intimate relationship between sphingolipid metabolism and thermal responses. However, the involvement of sphingolipids in heat resistance in the predator *C. lividipennis* has not been extensively studied.

In this research, we first identified two *CDase* homologous genes from the *C. lividipennis* genomic and transcriptomic databases. Then, the relative expression and phylogenetic analysis of CDases were conducted to understand the characteristics of these enzymes. Using the technology of RNA interference (RNAi), we clarified the biological roles of *ClAC* in thermal tolerance. Finally, transcriptome and lipid metabolome analyses were performed to reveal the potential regulatory mechanism.

## 2 Materials and methods

### 2.1 Insect rearing

*C. lividipennis* and their prey, BPH, were collected from a paddy field on the Zijingang campus of Zhejiang University, Hangzhou, China. The BPH population was maintained on susceptible rice seedlings of cv. Taichung Native 1 (TN1). *C. lividipennis* was reared in cages with fresh rice seedlings and sufficient prey. The environmental chamber parameters were set as 26 °C ± 1 °C, 70% ± 10% relative humidity, and a 14:10 h light: dark photoperiod, as described by Bai et al. (2022).

### 2.2 Sequence analysis

The homologous genes of *C. lividipennis CDase* were identified in the transcriptome data and genome data through local blast using *CDases* of *Homo sapiens*, BPH, and *Mus musculus* as queries. The phylogenetic analysis of ClAC and ClNC proteins was performed using MEGA X software (http://www.megasoftware.net/). A phylogenetic tree was constructed using the neighbor-joining and Poisson correction methods based on CDase protein sequences and setting the bootstrap value for 1,000 trials.

### 2.3 Sample collection

The sample collected for the stage-specific expression pattern analysis were eggs (*n* = 100), first instar nymphs (*n* = 50), second instar nymphs (*n* = 50), third instar nymphs (*n* = 15), fourth instar nymphs (*n* = 10), fifth instar nymphs (*n* = 10), and newly emerged females (*n* = 5) and males (*n* = 5). Tissues including the head, thorax, leg, integument, midgut, and fat body were dissected from third nymphs (*n* = 50). The third instar nymphs were collected after exposure under 26 °C and 38 °C for 6 h or 24 h (*n* = 5) to analyze the differently expressed genes induced by thermal stress. Three biological replicates were collected for each sample, then snap-frozen in liquid nitrogen and stored at −80 °C.

### 2.4 RNA isolation and quantitative real time polymerase chain reaction (qRT-PCR)

Total RNA was isolated using the TRIzol reagent (Invitrogen, Carlsbad, CA, United States), and cDNA was prepared following the manufacturer's protocol using *Evo M-MLV* RT Mix Kit with gDNA clean (Accurate Biotechnology). The qRT-PCR reaction was prepared with the SYBR Green premix *Pro Taq* HS qPCR Kit (Accurate Biotechnology) and performed using the CFX96 Real Time System (Bio-Rad Laboratories, Hercules, CA, United States). Each biological replicate had three technical replicates. The primers (Supplementary Table S1) employed for qRT-PCR were designed by Primer3 (v.0.4.0) (https://bioinfo.ut.ee/primer3-0.4.0/) based on the transcriptomic sequences. The standard curve method was employed to calculate the relative transcript levels. The housekeeping gene *GAPDH* was used as the internal reference gene.

### 2.5 RNA interference

*ClAC* and *Green Fluorescent Protein* (*GFP*) gene fragments were amplified using primers containing the T7 promoter (Supplementary Table S1). The cloned PCR products were used as templates to synthesize dsRNAs using the Thermo T7 Transcription Kit (TOYOBO). The DNA or RNA concentrations were measured by a NanoDrop 2000 Spectrophotometer (Thermo Fisher Scientific). The dsRNA products were diluted to 1000 μg/μL and stored at −80 °C for subsequent experiments. The dsRNA of *ClAC* (dsAC) and dsRNA of *GFP* (dsGFP) were separately microinjected into the mesothorax of third instar nymphs after anesthetizing with CO_2_. The treated third nymphs recovered in 30 min after microinjection were used for the following experiments. Three biological replicates (*n* = 5) were randomly collected for the measurement of RNAi efficiency.

### 2.6 Survival rate

The dsRNA-injected third instar nymphs were separately reared in 38 °C or 26 °C incubators for 12 h intervals to assess their survival rate. Six replicates were performed for each treatment. For each biological replicate, 12–16 injected third instar nymphs were transferred onto one BPH-oviposited rice plant, which was replaced in each glass tube every day to provide enough food resources for *C. lividipennis.*

### 2.7 Transcriptome sequencing

The dsAC-injected or dsGFP-injected third nymphs were reared at 38 °C for 24 h, and then sampled for the RNA extraction. Total RNA was extracted from 10 treated individuals for each sample. Three biological replicates were performed for both dsAC and dsGFP treatments. Illumina sequencing and cDNA library construction were carried out at Novogene (Beijing, China). The differentially expressed genes with padj<0.05 and |log2FoldChange|>0 were selected for the Gene Ontology (GO) and Kyoto Encyclopedia of Genes and Genomes (KEGG) analyses.

### 2.8 Lipidomic profiling

For each sample, 50 mg of dsAC-injected or dsGFP-injected third instar nymphs in total fresh weight were collected after rearing under 38 °C for 24 h. Six biological replicates were performed for both dsAC and dsGFP treatments. The lipid extraction and lipidomic profiling protocols were conducted at Metware Biotechnology laboratories (Wuhan, China). Significantly regulated metabolites between groups were determined by variable importance in projection (VIP) ≥ 1 and |log2 Fold Change| (|Log2FC|) ≥ 1.0.

### 2.9 Statistical analysis

The statistical values were shown as means ± standard error of the mean (SEM). One-way ANOVA and LSD test (*p*<0.05) were performed by Data Processing System (DPS) (Tang and Zhang, 2013). Student's t‐test was carried out in GraphPad Prism software (Swift, 1997).

## 3 Results

### 3.1 Expression patterns and phylogenetic analysis of *C. lividipennis* CDases

**FIGURE 1:**
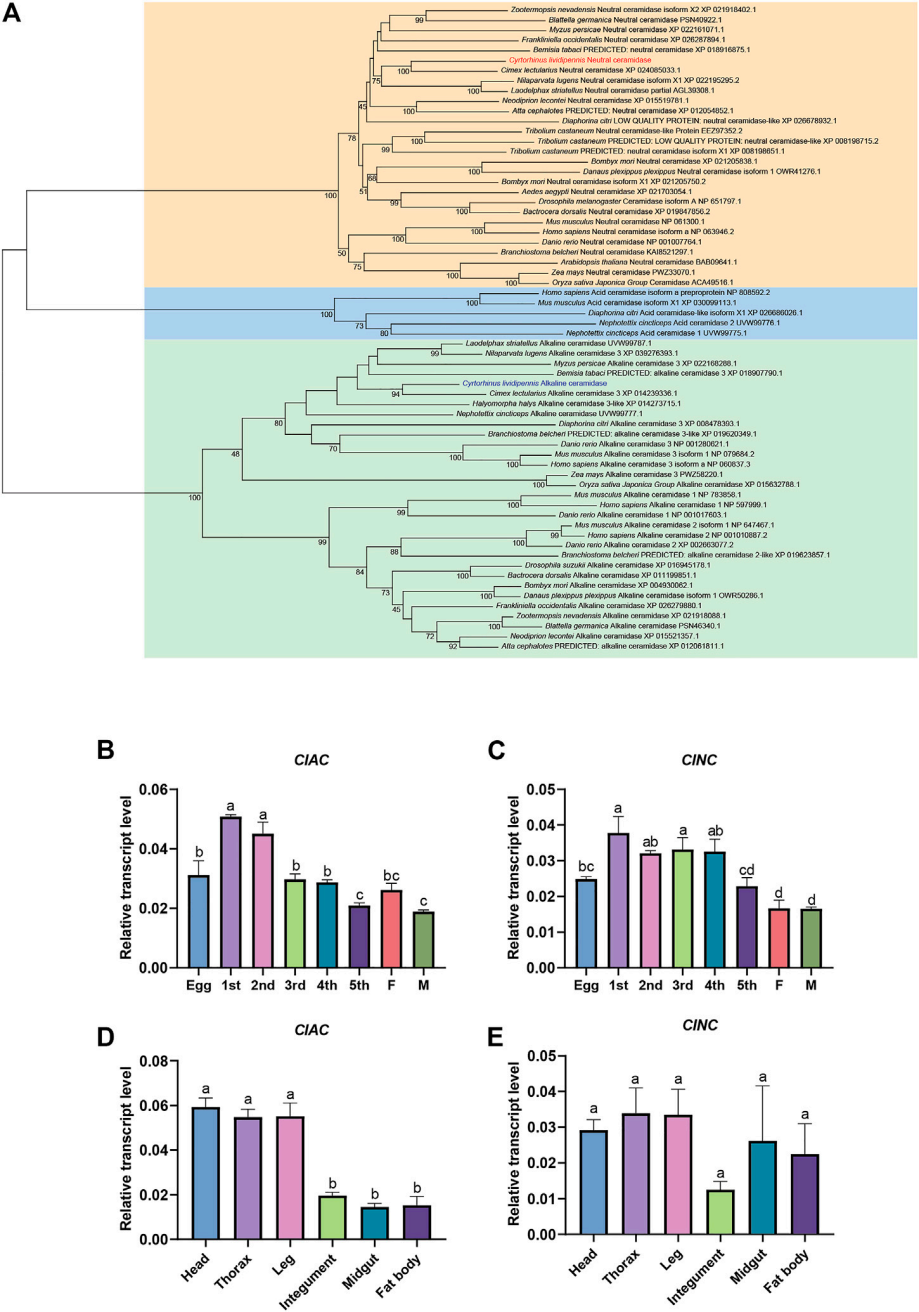
Expression patterns and phylogenetic analysis of *C. lividipennis* ceramidase. **(A)** Phylogenetic analysis of ClAC and ClNC with the homologous proteins. The neighbor-joining and Poisson correction methods were used to construct the phylogenetic tree based on ceramidase protein sequences. The bootstrap replication value was set to 1000. Relative transcript levels of *ClAC* **(B)** and *ClNC* **(C)** in eggs, nymphs, and adults. 1st, first instar nymph; 2nd, second instar nymph; 3rd, third instar nymph; 4th, fourth instar nymph; 5th, fifth instar nymph; F, female; M, male. Relative transcript levels *ClAC* **(D)** and *ClNC* **(E)** in different tissues. The error bar indicates the mean ± SEM of three independent biological replicates. Different letters indicate significant differences (*p* < 0.05).

To understand the CDases of *C. lividipennis*, we totally found two CDases from *C. lividipennis* (*ClAC* and *ClNC*). We analyzed their evolutionary relationships with CDases from humans, mice, plants, fishes, and other insects. The phylogenetic tree showed that *ClAC* was clustered into the aCDase group and closely related to the aCDases of the hemipteran insects *Cimex lectularius* and *L. striatellus*. Meanwhile, *ClNC* was clustered into the branch of nCDases and closely related to the hemipteran insect *C. lectularius* (Figure 1A). To further understand the biological function of these two CDases, the relative transcription levels of *ClAC* and *ClNC* across different developmental stages or tissues were investigated. The results showed that *ClAC* was expressed the highest in first and second nymphs, followed by eggs, third nymphs, fourth nymphs, females, and was the lowest in fifth nymphs and males. *ClNC* was expressed at a higher level in first to fourth nymphs than in eggs and fifth nymphs, and exhibited the lowest expression in adults (Figure 1B, C). These results indicated that *ClAC* and *ClNC* were more highly expressed in *C. lividipennis* early nymphs than adults. However, *ClAC* was higher expressed in the head, thorax, and legs than that the integument, midgut, and fat body, while *ClNC* showed no significant transcriptional differences between all studied tissues (Figure 1D, E). In conclusion, *C. lividipennis* had two CDases: one aCDase (ClAC) and one nCDase (ClNC), and both were conserved among hemipteran insect species. The transcription data on *ClAC* and *ClNC* suggested that *ClAC* and *ClNC* played different roles throughout the development of *C. lividipennis*.

### 3.2 The transcription level of *ClAC* was significantly increased under heat stress

**FIGURE 2:**
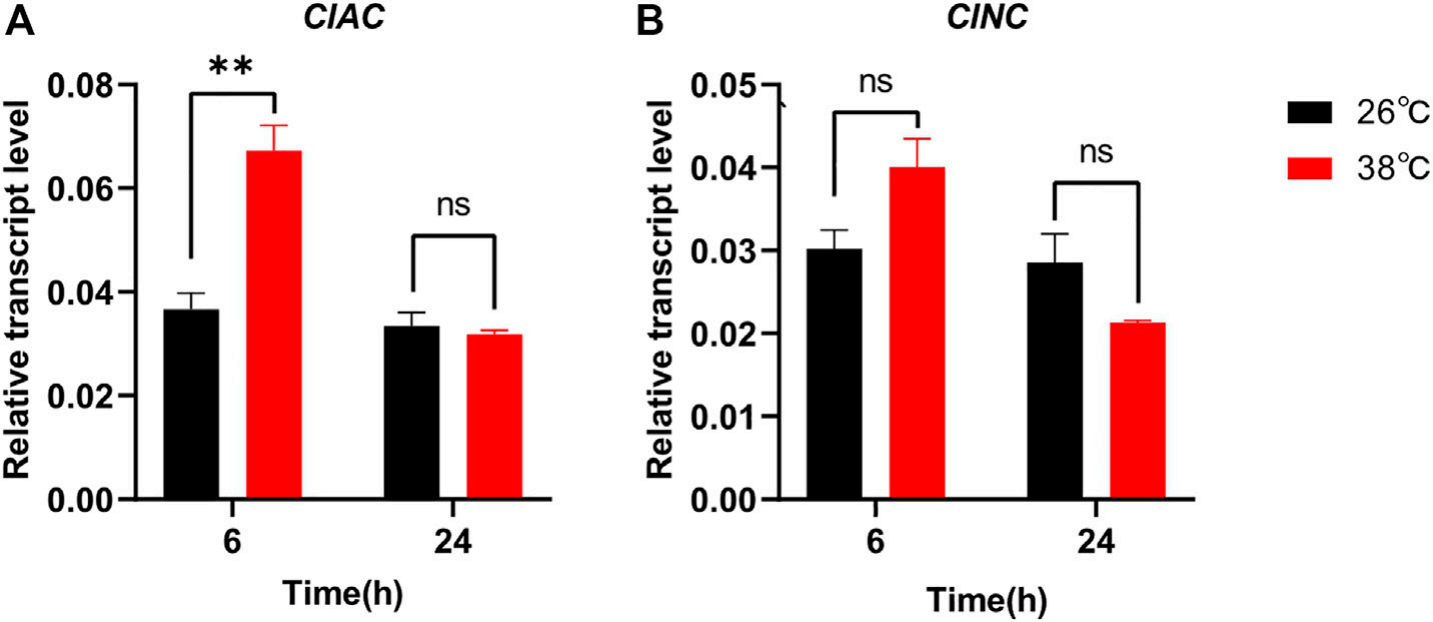
Relative transcript levels of *ClAC* **(A)** and *ClNC* **(B)** after thermal treatment. The gene transcription of *C. lividipennis* treated at 38 °C was shown by red bars and that at 26 °C by black bars. The error bar indicates the mean ± SEM of three independent biological replicates. Significant differences are indicated by “*” at *p*<0.05 and “**” at *p* < 0.01.

In order to reveal the relationship between sphingolipid metabolism and insect stress responses, we monitored the transcript levels of *ClAC* and *ClNC* after thermal treatment. The transcription of *ClAC* was first upregulated by two times after stressful heat treatment (38 °C) for 6 h, while it decreased to the normal level in the next 24 h (Figure 2A). Compared with the control group reared at the regular temperature (26 °C), the transcription of *ClNC* showed no significant differences under heat stress (38 °C) during all 24-hours treatments (Figure 2B). Meanwhile, the transcription levels of other sphingolipid genes, including *serine palmitoyltransferase* (*ClSPT*), *3-keto dihydrosphingosine reductase* (*ClKDSR*), *sphingolipid delta* *-desaturase* (*ClDES*), *sphingosine kinase* (*ClSK*), *sphingosine-1-phosphate phosphatase* (*ClS1PP*), *sphingomyelin synthase* (*ClSMS*), *sphingomyelinase* (*ClSMase*)*,* and *ceramide glucosyltransferase* (*ClCGT*)*,* were also monitored after heat treatment. The results showed that except for *ClAC*, *ClKDSR*, and *ClS1PP,* the sphingolipid genes had no significant transcriptional variation after the high-temperature treatment (Supplementary Figure S1). *ClKDSR* and *ClS1PP* were upregulated 1.9 times and 1.7 times, respectively, after stressful heat treatment (38 °C) for 6 h. These data indicated that *ClAC,* compared to *ClNC* or other sphingolipid genes, played more critical roles against heat stress in *C. lividipennis*.

### 3.3 Knocking down *ClAC* increased the survival rate of *C. lividipennis* under heat stress

**FIGURE 3:**
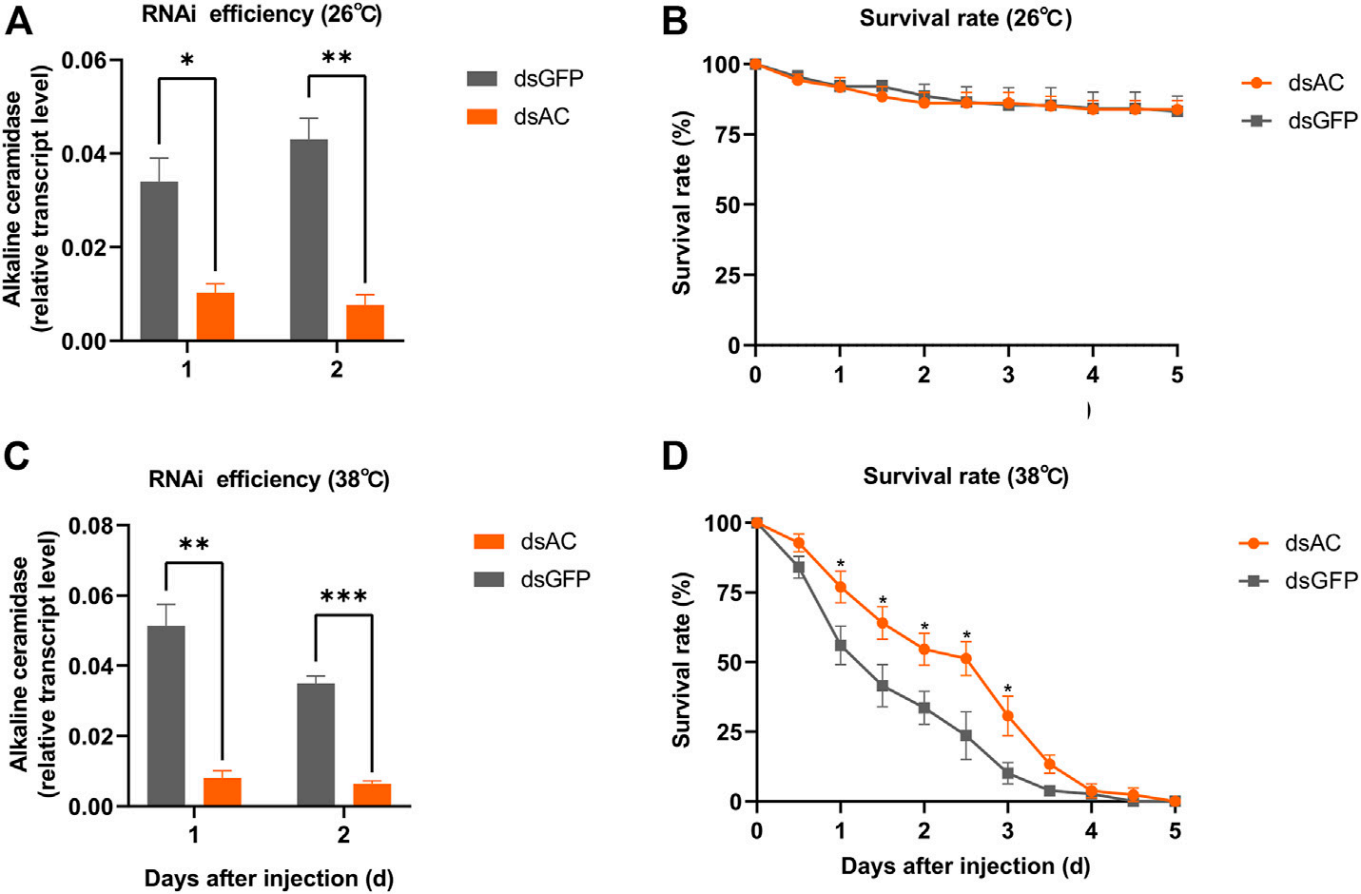
Roles of *ClAC* of *C. lividipennis* under heat stress. The relative transcription levels of *ClAC* at 26 °C **(A)** and 38 °C **(C)** after dsRNA injection. The error bar indicates the mean ± SEM of three independent biological replicates. The survival rate of third nymphs injected with dsAC (orange) and dsGFP (gray) at 12 h intervals below 26 °C **(B)** and 38 °C **(D)**. dsAC, dsRNA of *ClAC*; dsGFP, dsRNA of *GFP*. The error bar indicates the mean ± SEM of six independent biological replicates (*n* = 12–16 insects). Significant differences are indicated by “*” at *p* < 0.05, “**” at *p* < 0.01, and “***” at *p* < 0.001.

In order to investigate the roles of *ClAC* in heat stress response, we knocked down the expression of *ClAC* through RNAi. The relative transcript levels of *ClAC* were measured at 1 day or 2 days after the injection of dsRNA of *ClAC* (dsAC). Below 26 °C or 38 °C incubation, the injection of dsAC significantly inhibited the transcription of *ClAC*, and this transcriptional decline could last for two days (Figure 3A, C). Compared with the control group (the dsGFP-injected nymphs), nymphs with lower *ClAC* expression (dsAC-injected nymphs) showed no significant difference in the survival rate at 26 °C (Figure 3B). When the rearing temperature was 38 °C, the dsAC-injected *C. lividipennis* had higher survival rate than the dsGFP-injected group (Figure 3D). This indicated that lower *ClAC* expression increased the survival rate of *C. lividipennis* nymphs under heat stress. However, the mechanism through which *ClAC* mediates the heat stress responses in *C. lividipennis* is still unknown.

### 3.4 Transcriptome analysis

**FIGURE 4:**
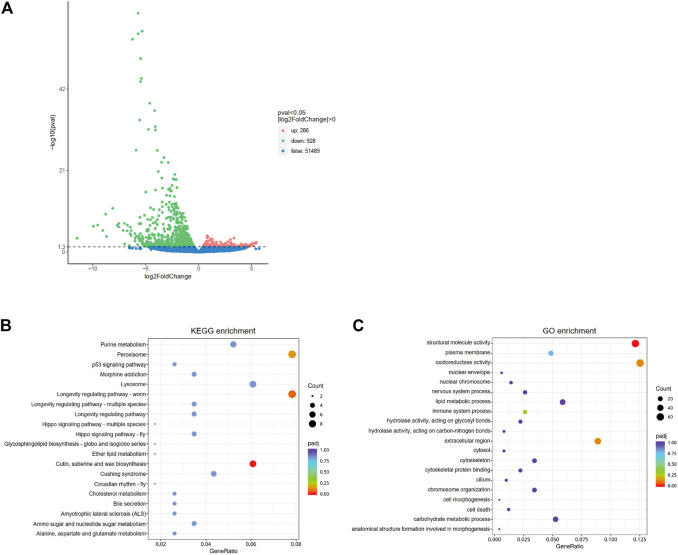
Analysis of comparative transcriptome data. **(A)** Volcano plot of differentially expressed genes (DEGs) between dsAC and dsGFP treatments. The red, green, and blue dots represent significantly upregulated genes, significantly downregulated genes, and non-significantly different genes, respectively. DEGs are filtered based on the criteria of padj<0.05 and |log2FoldChange|>0. The most enriched KEGG pathway **(B)** and GO terms **(C)** of DEGs. The sizes of the dots represent the number of DEGs, and the colors represent the enrichment level of DEGs.

In order to further understand the mediating role of *ClAC* in heat responses, the transcriptome of third nymphs at the first day after dsRNA injection was analyzed. A total of 1214 differentially expressed genes (DEGs) were detected between the two groups. Compared with dsGFP-injected nymphs, the transcription of 286 genes was upregulated, while that of 928 genes were downregulated in dsAC-injected third nymphs (Figure 4A). KEGG enrichment revealed that the DEGs were mainly involved in peroxisome (nine DEGs), cutin, suberine, and wax biosynthesis (seven DEGs), and longevity regulating pathways (nine DEGs) (Figure 4B). In the longevity regulating pathway, the transcription of *catalase* (*CAT*), *alcohol-forming fatty acyl-CoA reductase* (*FARD-1*), and *stearoyl-CoA desaturase* (*FAT-6*) were significantly varied (Supplementary Figure S2). GO enrichment analysis showed that DEGs were mostly clustered in the structural molecule activity, oxidoreductase activity, and extracellular region (Figure 4C). To reveal the relationship between *ClAC* and oxidoreductase, we quantified the transcript levels of oxidoreductase-related genes including two *CAT* (*ClCAT1* and *ClCAT2*), two *glutathione peroxidases* (*ClGPX1* and *ClGPX2*), five *peroxidases* (*ClPOD1 to ClPOD5*), and five *superoxide dismutases* (*ClSOD1 to ClSOD5*). The transcription of *ClCAT1* instead of other genes was significantly increased by 2.2 times in the dsAC-injected third nymphs (Supplementary Figure S3). Moreover, we quantified the relative transcription levels of other sphingolipid genes after knocking down *ClAC*. The qPCR results showed that the transcript level of *ClKDSR* and acid sphingomyelinase *2* (*ClaSMase2*) were upregulated by 1.9 times and 1.3 times, respectively, while other sphingolipid genes had no significant transcription difference between the dsAC-injected nymphs (Supplementary Figure S4).

### 3.5 Lipid profiling analysis

**FIGURE 5:**
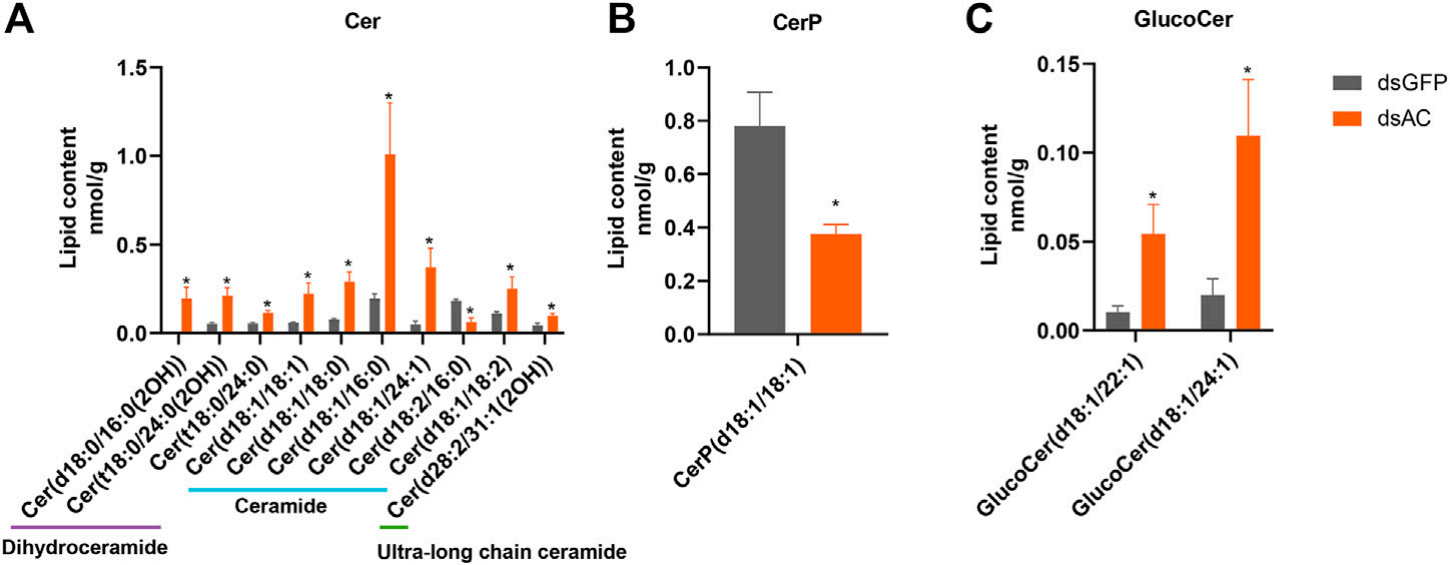
Sphingolipid levels after dsAC- and dsGFP-injection. The levels of ceramides **(A)**, ceramide-1-phosphate **(B),** and glucoceramide **(C)** in *C. lividipennis* nymphs after dsAC- and dsGFP-injection. dsAC-injected group, orange column; dsGFP-injected group, gray column. Significant differences are shown by “*” at VIP (VIP ≥ 1) and absolute log2FC (|log2FC| ≥ 1.0).

Lipid profiling was conducted after the transcriptional inhibition of *ClAC* to investigate the metabolic functions of *ClAC* and reveal the network between sphingolipids and other metabolites. A total of 32 differential lipid metabolites (DLMs) were detected, of which 19 DLMs were significantly increased and 13 DLMs were significantly decreased (Supplementary Figure S5A). The KEGG enrichment of DLMs showed that 13 of the 32 DLMs were enriched in the sphingolipid pathway (41%) (Supplementary Figure S5B). These sphingolipids included 10 ceramides (Cer), 1 ceramide-1-phosphate (CerP), and 2 glucosylceramides (GlucoCer). Cer(t18:0/24:0(2OH)) and Cer(t18:0/24:0) were upregulated by 3.9 and 2.1 times, respectively. Ceramides, including Cer(d18:1/18:1), Cer(d18:1/18:0), Cer(d18:1/16:0), Cer(d18:1/24:1), and Cer(d18:1/18:2) were increased by 3.8, 3.8, 5.2, 7.5, and 2.3 times, respectively. The ultra-long chain ceramide Cer(d28:2/31:1(2OH)) was significantly increased by 2.2 times. A trace amount of dihydroceramide Cer(d18:0/16:0(2OH)) (0.23 nmol/g) was detected in dsAC-injected nymphs after high-temperature treatment, while none were observed in the dsGFP-injected nymphs. The levels of Cer(d18:2/16:0) and CerP(d18:1/18:1) were significantly decreased by 41% and 48%, respectively. Meanwhile, GlucoCer(d18:1/22:1) and GlucoCer(d18:1/24:1) were significantly increased by 5.2 and 4.6 times, respectively (Figure 5). In conclusion, most ceramides were increased after *ClAC* inhibition, and *ClAC* mainly mediated the levels of C18 sphingo-based ceramides and their glycosylation derivatives (GlucoCers) in the response to heat stress.

## 4 Discussion

CDases are classified according to their optimal pH for enzymatic activity (Mao et al., 2001). The types of CDase vary among different organisms. Therefore, it is necessary to investigate the CDase categories for their functional analysis. Five CDase homologs genes have been identified from humans, including one aCDase, one nCDase, and three alCDases (Coant et al., 2017). Two CDases were found in the hemipteran insect *Nephotettix cincticeps* (Zhang et al., 2021). Similar to *C. lividipenni*s, the nCDase and alCDase of *Drosophila* (CDase and Dacer) were reported, whereas no aCDase homologs have been identified (Yoshimura et al., 2002; Acharya and Acharya, 2005; Yuan et al., 2011). The nCDase and alCDase were also reported in the hemipteran insect *Laodelphax striatellus* (Zhou et al., 2013; Zhang et al., 2021). The phylogenetic analysis demonstrated that CDases are highly conserved between insects, while the biological function of different type CDases vary among different insect species. The alCDase of *Drosophila* (*Dacer*) was highly expressed in the pupal stage and significantly affected the longevity of *Drosophila* (Yang et al., 2010; Zhang et al., 2019). The nCDase of *Tribolium castaneum* showed high expression in adults, but its biological functions had not been demonstrated (Zhou et al., 2011).The nCDase of BPH was highly expressed in female adults and played essential roles in the reproduction of the insect (Shi et al., 2018; Shi et al., 2021). Different from BPH or *Tribolium castaneum*, both *ClNC* and *ClAC* had high expression in the early nymph stages. The relative transcript levels of genes involved in the *de novo* biosynthetic pathway and sphingomyelinase pathway were tested to observe ceramide metabolism across *C. lividipennis* different developmental stages (Supplementary Figure S6). In the *de novo* biosynthetic pathway, *ClSPT2*, *ClKDSR*, and *ClCS* were highly expressed in eggs, followed by nymphs and females, and exhibited the lowest levels in males. *ClDES* was highly expressed in nymphs, while was lowest in eggs and adults. It suggested high levels of ceramides might synthesize from the *de novo* biosynthetic pathway. In the sphingomyelinase pathway, sphingomyelinases (*ClnSMase*, *ClaSMase1*, and *ClaSMase2*) hydrolyze sphingomyelin to ceramide, while sphingomyelin synthases (*ClSMS*) synthesize ceramide into sphingomyelin (Gault et al., 2010). The relative transcription data showed that *ClnSMase* was highly expressed in eggs and nymphs, and lowest in adults. Coordinated with *ClAC* and *ClNC*, the expression of *ClaSMase1* and *ClaSMase2* were highest in the first nymph, decreased from the first to fifth nymph, and lowest in eggs and adults, indicating ClAC or ClNC might hydrolyze the ceramides produced by ClSMases. Moreover, *ClSMS* was also higher expressed in first nymphs than in other development stages. These results showed that the sphingolipid genes are more highly expressed in early nymph stages than in the adults, indicating the ceramide metabolism was more active in *C. lividipennis* nymphs. In order to maintain a balance of ceramide metabolism, *ClAC* and *ClNC* were supposed to be highly expressed to degrade the excessive ceramides in nymphs. In BPH, the transcript level of nCDase was upregulated under heat stress and knocking down of nCDase increased the female survival rate (Shi et al., 2018). Meanwhile, our results showed that *ClAC* instead of *ClNC* responded to the thermal threat in *C. lividipenni*s, suggesting that *ClNC* played different roles together with *ClAC*, and the function of *ClNC* need to be further investigated.

In the present study, we attempted to explain how *ClAC* mediated the high temperature resistance in *C. lividipenni*s through transcriptome and lipodomics analysis. For the transcription level, the inhibition of *ClAC* directly mediated the transcription of longevity-related genes and other sphingolipid metabolism-related genes (*ClKDSR* and *ClaSMase2*) to control the hyper thermal responses in *C. lividipenni*s. As previously reported, thermal stress induces the generation of reactive oxygen species (ROS), including superoxide anion (O2^−^), hydroxyl radicals (^−^OH), and hydrogen peroxide (H_2_O_2_), which can cause oxidative damages and lead to cell death in living organisms (Cui et al., 2011). Antioxidants are necessary to reduce the content of ROS and improve the cellular fitness. Enzymatic antioxidants, such as SOD, CAT, POD, and GPX, have been identified as ROS scavengers (Wang et al., 2001). CAT as one of the primary antioxidant enzymes protects organisms from oxidative damage by catalyzing hydrogen peroxide into water and oxygen (Wang et al., 2001). CAT was also reported to play important roles in the thermal tolerance of insects. Suppressing *CAT* significantly reduced the survival rate of *Myzus persicae* under heat stress (Li et al., 2021). In the whitefly *Bemisia tabaci*, CAT help to adapt to high temperature by scavenging excessive ROS (Liang et al., 2022). The transcript level and activities of *CAT* were enhanced to reduce the production of ROS in mutant *Dacer*, aiding resistance to paraquat-induced oxidative stress (Yang et al., 2010; Zhang et al., 2019). Therefore, we speculated that the high expression of *CAT* after *ClAC* inhibition in *C. lividipenni*s might enhance the antioxidant ability of nymphs against the abundant ROS caused by heat stress. However, sphingolipids as the non-enzymatic antioxidants, especially for the ceramides, have been demonstrated to mediate oxidoreductase and maintain cellular redox homeostasis by regulating antioxidant enzymes (García-Caparrós et al., 2021). C2 ceramide inhibited ROS production and cell death in H_2_O_2_-treated rat primary astrocytes by increasing the expression of phase II antioxidant enzymes, including heme oxygenase-1 (HO-1), NAD(P)H:quinine oxidoreductase 1 (NQO1), and superoxide dismutase (SOD), attesting to the therapeutic potential of C2 ceramide for various oxidative stress-associated diseases (Jung et al., 2016). The treatment of ceramide and ascorbic acid increased the activities of POD and SOD, thus significantly reduced the oxidative damages and maintained the storage quality of strawberries (Zhao et al., 2019). Taken together, the change in ceramide levels after *ClAC* inhibition might mediate the expression of *CAT* and affect the survival rate of *C. lividipenni*s under heat stress. Moreover, the *ClKDSR* and *ClaSMase2* closely regulated the ceramide levels through *ClAC* than other sphingolipid gene in *C. lividipenni*s.

For the metabolite level, ceramides are not only the essential structural compositions of plasma membranes (Fabri et al., 2020), but also the signaling molecules mediating cellular growth, apoptosis, or death (Okazaki et al., 1998). Ceramides were demonstrated to play important roles in stress tolerance. The loss of the ceramide synthase gene *hyl-2* made *Caenorhabditis elegans* more sensitive to heat shock and anoxia, which indicated that the specific ceramides synthesized by *hyl-2* were required for the stress responses (Menuz et al., 2009). The C16-ceramide synthesized by ceramide synthase 6 had anti-apoptotic roles in tumor cells when encountering endoplasmic reticulum (ER) stress (Senkal et al., 2010). The variation in ceramide species and concentration affected the plasma membrane fluidity. Due to the reduced ceramide levels after ceramide transfer protein (CERT) inhibition, the vesicles were loosely packed, less homogenous, and non-discrete in the plasma membrane of *Dcert* (Rao et al., 2007). The increased fluidity of plasma membrane made *Dcert* mutants more susceptible to oxidative damage (Rao et al., 2007). In addition, ceramides with different acyl-chains had different impact on the membrane biophysical properties. Sandra *et al.* found that saturated ceramides (C16-, C18-, and C24-) were preferred to form gel domains over unsaturated ceramides (C18:1- and C24:1-) in phosphatidylcholine model membranes, and the gel domain formed by C16-ceramide was larger and more stable than that formed by C18-ceramide (Pinto et al., 2011). In the present study, the increased ceramide levels proved the hydrolysis function of *ClAC* as the CDase in *C. lividipennis*. Saturated Cer (d18:1/16:0) was the most abundant species and unsaturated Cer (d18:1/24:1) was the most significantly increased species after *ClAC* inhibition, indicating that Cer (d18:1/16:0) and Cer (d18:1/24:1) were the dominant ceramide species catalyzed by *ClAC*. Cer (d18:1/16:0) reached the highest level compared to other ceramides and might be associated with plasma membrane fluidity. Thus, we proposed that this molecule might play an important role in enhancing the cell membrane stability and in turn upregulate the resistance to oxidative stress.

Most entomology studies have profiled CDases in the model insect *D. melanogaster* and other insect pests such as BPH. However, the CDases profiles have not been studied in natural enemy insects. We found two CDases, *ClAC* and *ClNC*, in *C. lividipennis* and demonstrated that *ClAC* is essential in heat stress response. Metabolically, *ClAC* mediated the ceramide levels and directly regulated the oxidoreductase-related genes at the transcription level. Our results suggested that inhibiting *ClAC* increased the survival rate of *C. lividipennis* nymphs, which was regulated by the alteration of ceramide levels and *ClAC* downstream genes. This study validated the *ClAC* functions and provides valuable information for improving the heat stress tolerance of *C. lividipennis* and other natural enemies of pest insects. Further study should be conducted to construct a sphingolipid gene network to enhance sphingolipid applications in green and sustainable biological pest control.

## Data Availability

The datasets presented in this study can be found in online repositories. The names of the repository/repositories and accession number(s) can be found at: https://www.ncbi.nlm.nih.gov/bioproject/PRJNA932139.
