# Supplementary figures and images for: Alkaline ceramidase (ClAC) inhibition enhances heat stress response in Cyrtorhinus lividipennis (Reuter)
Source: Front Physiol. 2023 May 9;14:1160846. doi: 10.3389/fphys.2023.1160846 (PMC10206425; doi:10.3389/fphys.2023.1160846)

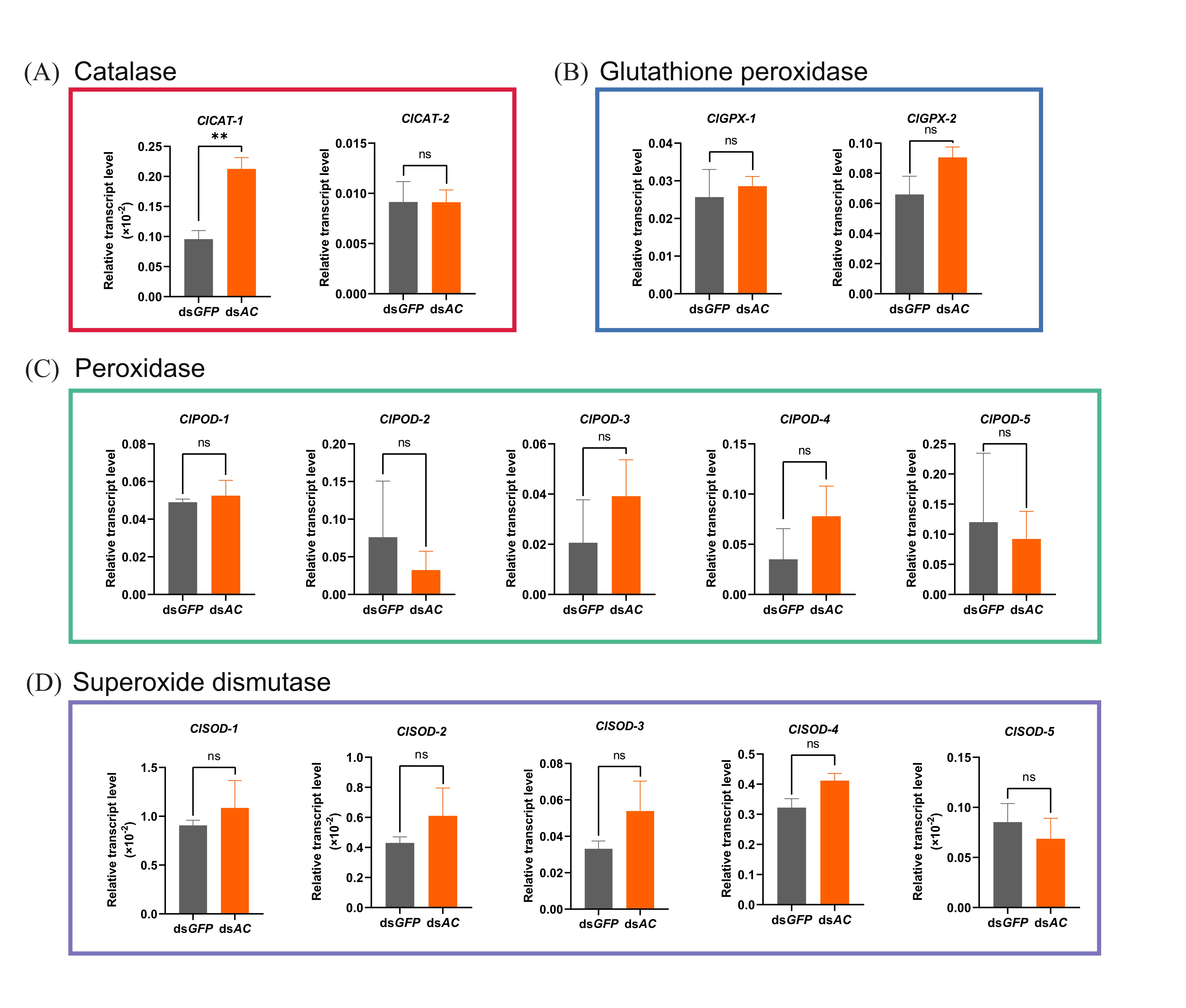

Supplement: Supplementary file 1 [file Image3.JPEG]

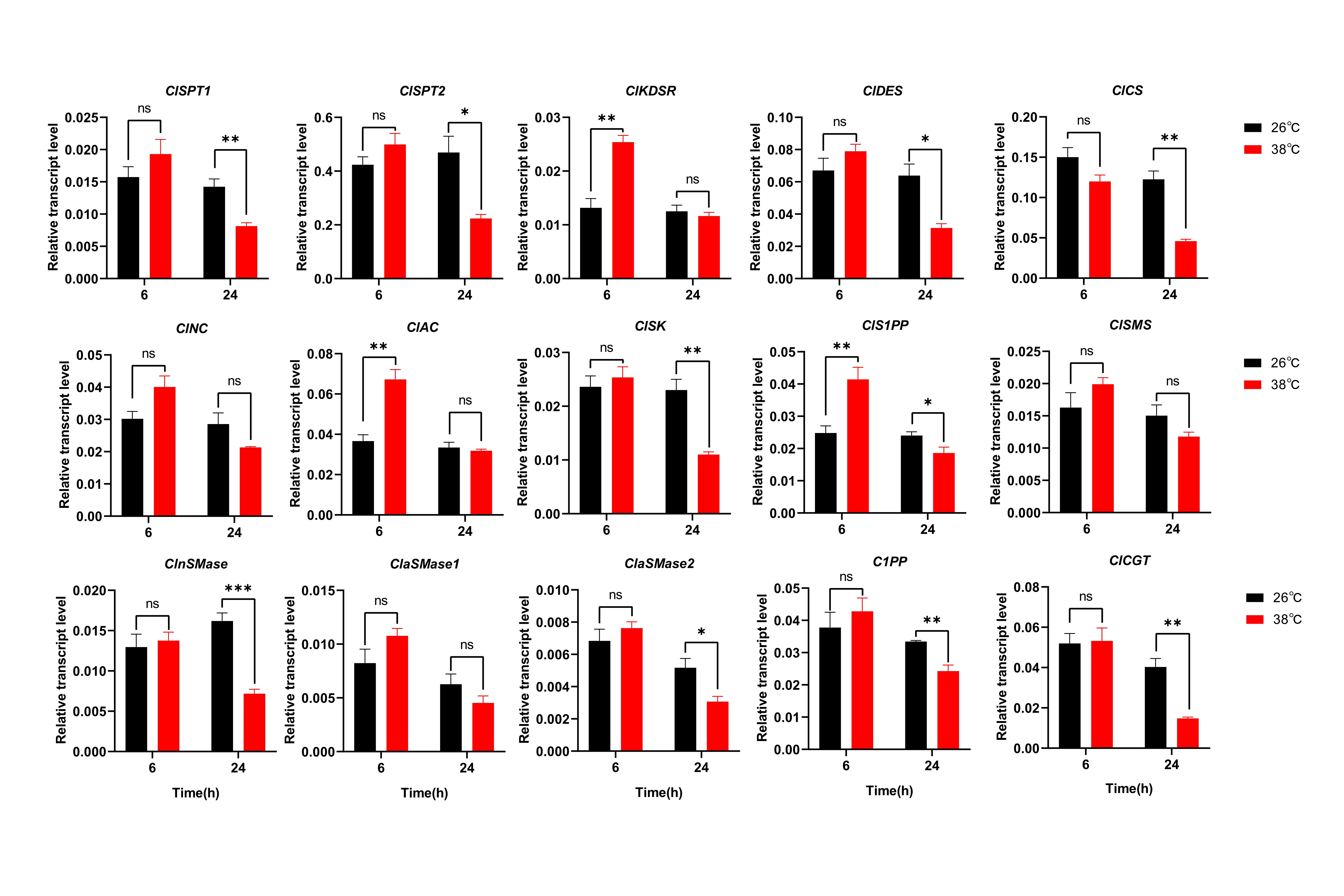

Supplement: Supplementary file 2 [file Image1.JPEG]

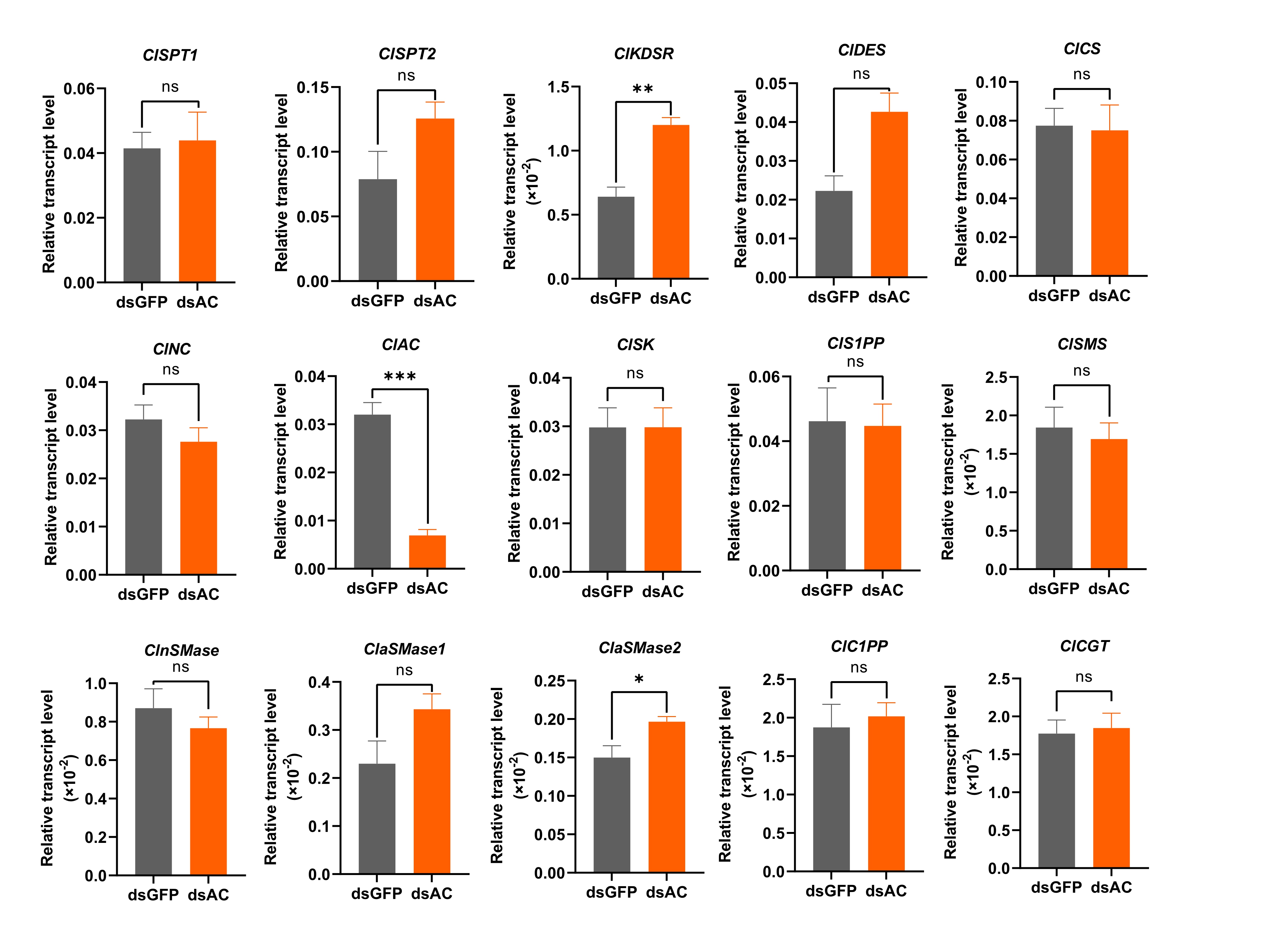

Supplement: Supplementary file 3 [file Image4.JPEG]

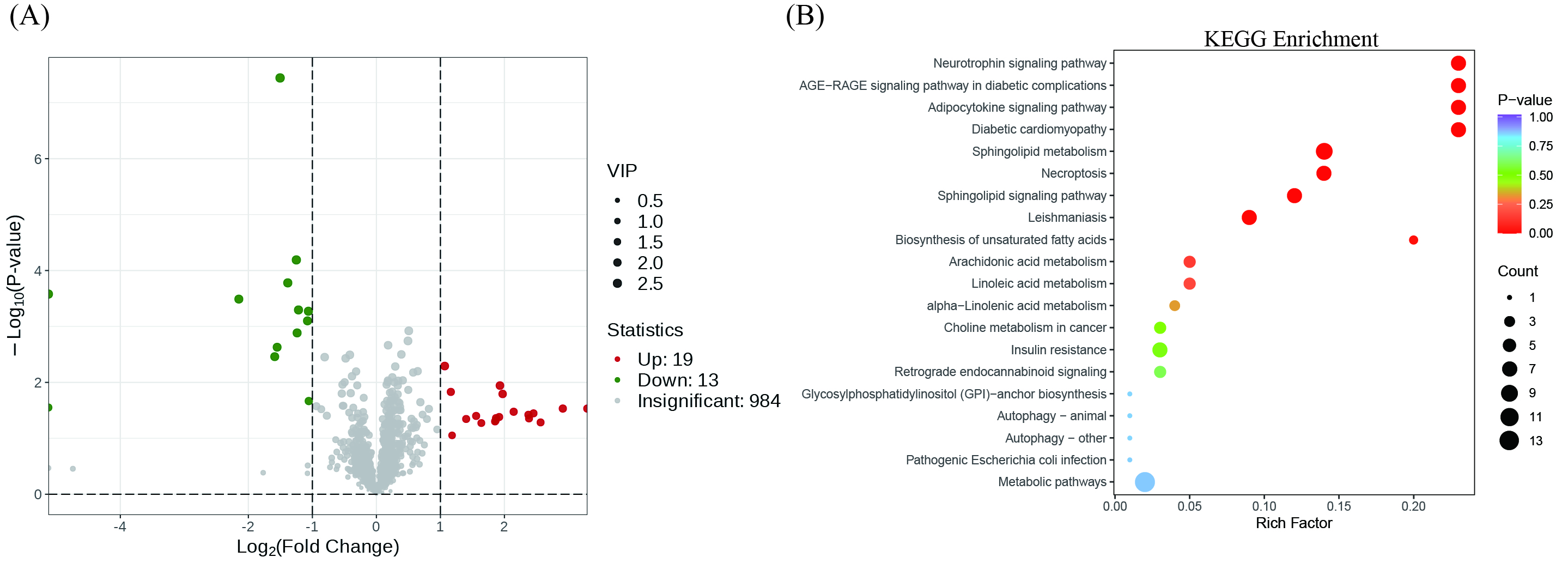

Supplement: Supplementary file 4 [file Image5.JPEG]

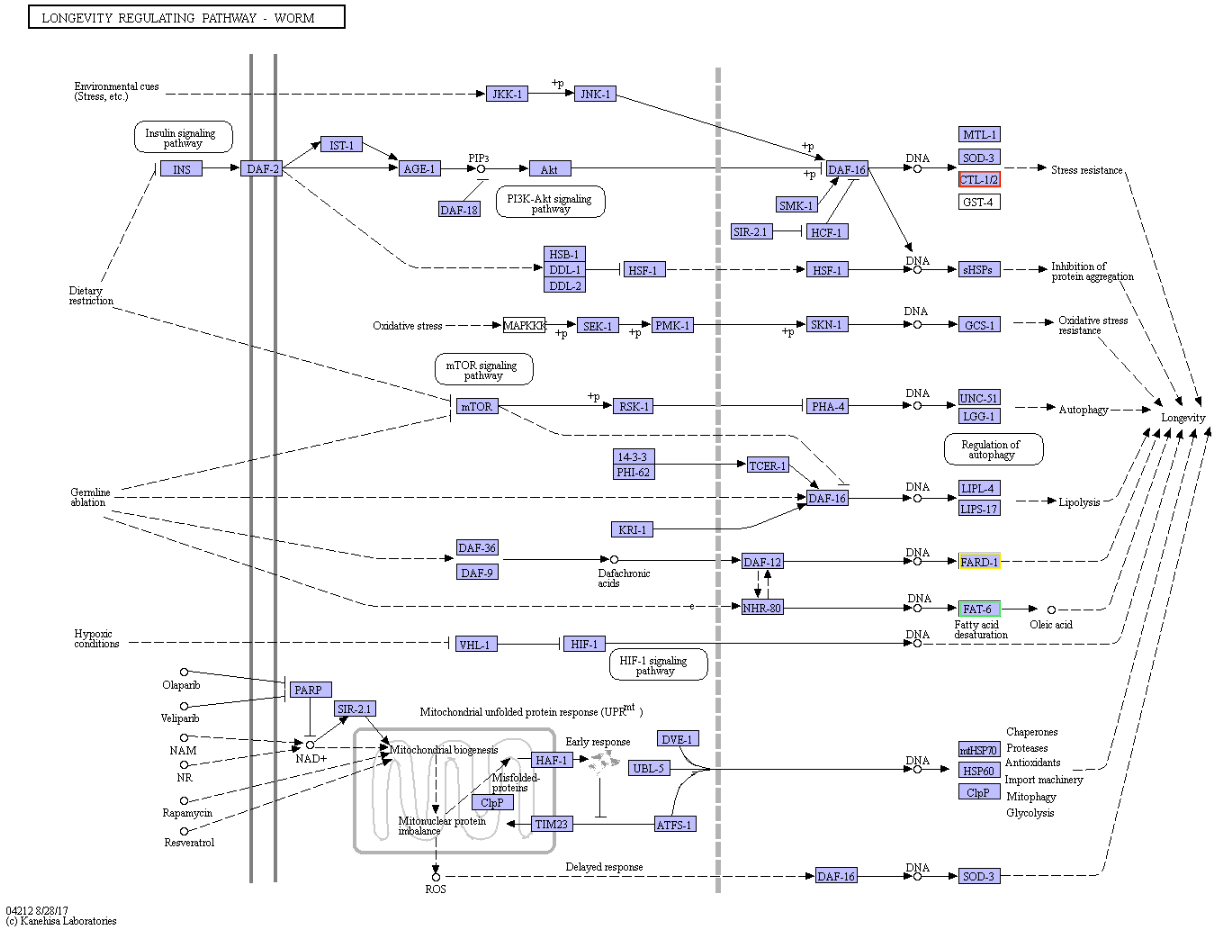

Supplement: Supplementary file 5 [file Image2.PNG]

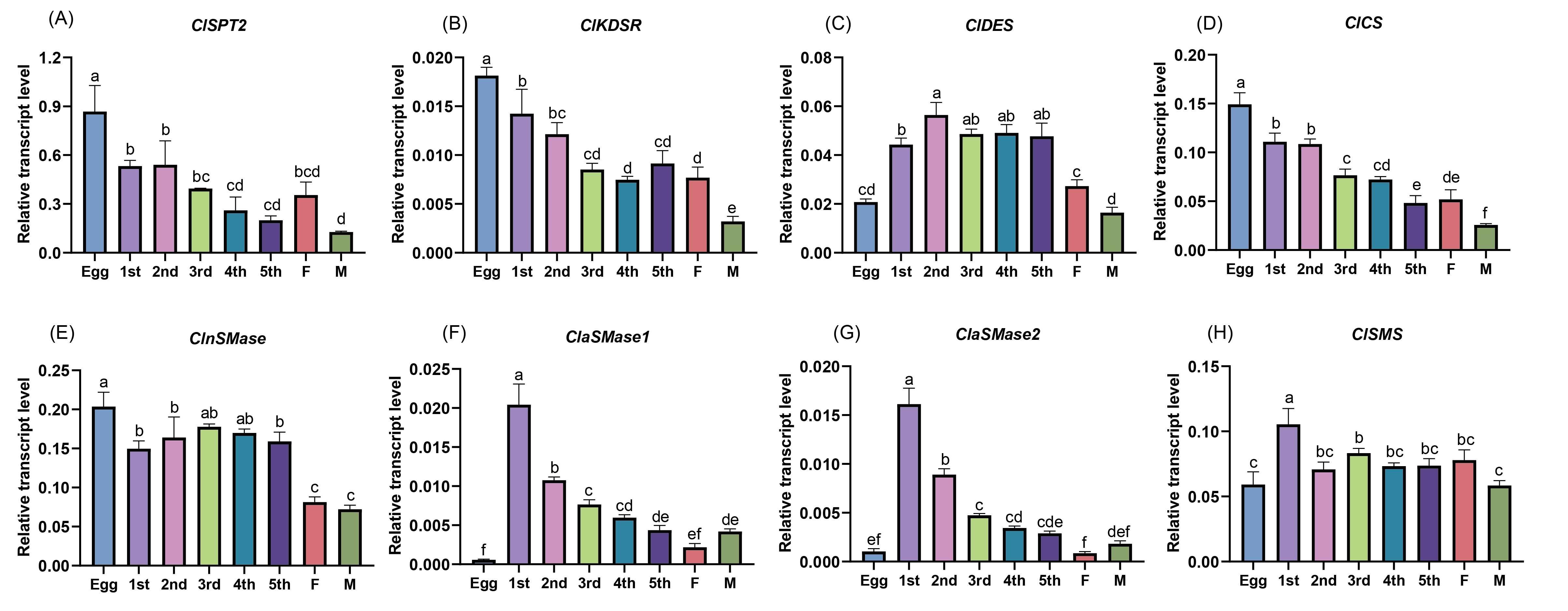

Supplement: Supplementary file 7 [file Image6.JPEG]
