# Supplementary material for: Alkaline ceramidase (ClAC) inhibition enhances heat stress response in Cyrtorhinus lividipennis (Reuter)
Source: Front Physiol. 2023 May 9;14:1160846. doi: 10.3389/fphys.2023.1160846 (PMC10206425; doi:10.3389/fphys.2023.1160846)
Supplement: Supplementary file 6 [file DataSheet1.docx]

Supplementary Material

Alkaline ceramidase (*ClAC*) inhibition enhances heat stress response in *Cyrtorhinus lividipennis* (Reuter)

**Min Chen, Xiao-Xiao Shi*, Ni Wang, Chao Zhang, Zhe-Yi Shi, Wen-Wu Zhou, Zeng-Rong Zhu***

*** Correspondence:**

Zhu, Zeng-Rong: [zrzhu@zju.edu.cn](mailto:zrzhu@zju.edu.cn)

Shi, Xiao-Xiao: shixiao6656@163.com

# Supplementary Figures Legend

Supplementary Figure 1. Effect of heat stress on the dynamic changes of *C. lividipennis* sphingolipid metabolism pathway genes transcription. Bar indicate mean ± SEM of three independent biological replicates. Significant differences are shown with "*" at *p*<0.05, "**" at *p*<0.01, "***" at *p*<0.001.

Supplementary Figure 2. Relative expression of oxidoreductases by qRT-PCR assay. The relative transcript levels of catalases (A), glutathione peroxidases (B), peroxidases (C) and superoxide dismutases (D) after dsAC- (orange) or dsGFP- (gray) injection. The error bars indicate the mean ± SEM of three independent biological replicates. Significant differences are shown with "*" at *p*<0.05, "**" at *p*<0.01, "***" at *p*<0.001.

**Supplementary Figure 3.** Relative transcript levels of genes involved in sphingolipid metabolism after dsAC- (orange) or dsGFP- (gray) injection. The error bars indicate mean ± SEM of three independent biological replicates. Significant differences are shown with "*" at *p*<0.05, "**" at *p*<0.01, "***" at *p*<0.001.

Supplementary Figure 4. Longevity regulating pathway. Red border indicates upregulated genes. Green brder indicates downregulated genes. Yellow border indicates both upregulated and downregulated genes.

**Supplementary Figure 5.** Analysis of lipid metabolomic profiling. (A) Volcano plot of differential lipid metabolites (DLMs) between dsAC and dsGFP treatments. Red, green and grey dots represent significantly increased metabolites, significantly decreased metabolites and non-significantly different lipid metabolites, respectively. DLMs between groups were determined by VIP (VIP ≥ 1) and absolute log2FC (|log2FC| ≥ 1.0). (B) KEGG enrichment scatter plot. The sizes of the dots represent the number of DLMs and the colours represents the enrichment level of DLMs.

**Supplementary Figure 6.** Expression patterns of *C. lividipennis* sphingolipid enzymes. Relative transcript levels of *ClSPT2* (A), *ClKDSR* (B), *ClDES* (C), *ClCS* (D), *ClnSMase* (E), *ClaSMase1* (F), *ClaSMase2* (G), and *ClSMS* (H) in eggs, nymphs and adults. 1st, first instar nymph; 2nd, second instar nymph; 3rd, third instar nymph; 4th, fourth instar nymph; 5th, fifth instar nymph; F, female; M, male. The error bar indicates the mean ± SEM of three independent biological replicates. Different letters indicate significant differences (*p*<0.05).

# Supplementary Tables

Supplementary Table 1. Specific primers used in this research

| **Purpose** | **Prime name** | **Prime sequence（5‘-3’）** |
| --- | --- | --- |
| **Sphingolipid metabolism pathway** | qAC-F | GGAAGCCAACGTCCACTATT |
|  | qAC-R | CGCTTCTCGAAATTTTGTTTG |
|  | qNC-F | CCCAGTCAGCATCTGTAGCA |
|  | qNC-R | AGACTGTAAAGCGGGGGTCT |
|  | qKDSR-F | GGTGGATCGTCTGGTATTGG |
|  | qKDSR-R | AACAGGAAAAGATGCGTTGC |
|  | qDES-F | GCAATACACGAAATCGCTCA |
|  | qDES-R | CTCCTTGGTACCGATGGTGT |
|  | qCS-F | ATGTTACTTGGGCGGACTTG |
|  | qCS-R | TCTAATTCCCAACGCGATTC |
|  | qS1PP-F | CGTCCGAGGAAGACGATTTA |
|  | qS1PP-R | CGTTCCAAAACCAAAAAGGA |
|  | qSPT1-F | TGGGTGACGTCATTGTGTTT |
|  | qSPT1-R | CTCCTACGGATTTTCCACCA |
|  | qSPT2-F | CGAAAAGCTTACTGCCGAGT |
|  | qSPT2-R | AAGACCAAGGATGAGCGAAG |
|  | qSK-F | TGGAGATCATGGACATCGAA |
|  | qSK-R | ACGTTGCCGTTCATCTCTTT |
|  | qCGT-F | CTCGCAAATACCCGAGTTCT |
|  | qCGT-R | GGAAGCACTTGATCGAGGAG |
|  | qSMS-F | AGAACGAGAGGCGATTCGTA |
|  | qSMS-R | CTCCCGCCTCTTCCTCTACT |
|  | qnSMase-F | CGTTCCAATGGTTCTTGGTT |
|  | qnSMase-R | TCGGTGTAAATTCGTGCTTG |
|  | qaSMase1-F | GCCATCTGGGACGAAGAATA |
|  | qaSMase1-R | GCCATCCAGTACCTTCCAAA |
|  | qaSMase2-F | GGTCCTGGATTACACGCAGT |
|  | qaSMase2-R | GTGCTCTGTGAACGTGTTGG |
|  | qC1PP-F | CGTCTCCGTCCTCATTTCAT |
|  | qC1PP-R | CGAGTGTCCTGAAGGGAAAG |
| **Oxidoreductases** | qCAT-1-F | ATCGCTACATGGACGGTTTC |
|  | qCAT-1-R | TTGTTGACGCGAGAAGATTG |
|  | qCAT-2-F | GCTGGTGTGGATCCTGACTT |
|  | qCAT-2-R | CCATGGCATTTTCTTTGCTT |
|  | qGPX-1-F | TGCCGTATTGGGTGACTCTT |
|  | qGPX-1-R | TTGGTGTTGTTCCAGCCATT |
|  | qGPX-2-F | TCGATGTAAATGGCGAGACA |
|  | qGPX-2-R | TGATGAGTGCCTCGAAACTG |
|  | qPOD-1-F | TCGACTGCAACAAGAAATGC |
|  | qPOD-1-R | TGGCGTAAGTTGTGAAGCAG |
|  | qPOD-2-F | CACACGAAGAATGAGCCTGA |
|  | qPOD-2-R | CAGAAGGGAAAGACCTGACG |
|  | qPOD-3-F | GGTCCTTCCAGACCATGAGA |
|  | qPOD-3-R | GGATCGAAATTGTTGGCATT |
|  | qPOD-4-F | GGTGGACCATTAGGACATGG |
|  | qPOD-4-R | CAAGGAGAAGCTCTCCAACG |
|  | qPOD-5-F | ACTTCCTGGAGCTCGTTTGA |
|  | qPOD-5-R | TTGGTTAGCGTAGGGTTTGG |
|  | qSOD-1-F | AGGCACCGACTCAGAAGAAA |
|  | qSOD-1-R | CCACAAGACACCTTCCCACT |
|  | qSOD-2-F | ACCTTTCTCACGGCTCAAGA |
|  | qSOD-2-R | CTGTCCCATCCTCACCAACT |
|  | qSOD-3-F | CCGATTAACGCCATTTGTGT |
|  | qSOD-3-R | GAATTCGTGGATGTGGAACC |
|  | qSOD-4-F | ACTTCAATCCCCACAGCAAG |
|  | qSOD-4-R | AGGTCACCACAGAGCGAGAT |
|  | qSOD-5-F | CAGTCACGAGGGCTACACAA |
|  | qSOD-5-R | GTGGCGTGATGTTTTTGATG |
| **Housekeeping gene** | qGAPDH-F | ACGTCTCTGTCGTCGACCTT |
|  | qGAPDH-R | ACTTGATCGTCGGTGTAGCC |
| **RNAi** | dsAC-F | taatacgactcactatagggGTTTCACATGACGCTGCTGT |
|  | dsAC-F | taatacgactcactatagggAATATGTGCCATAGCCTGCC |
|  | dsGFP-F | taatacgactcactatagggAAGTTCAGCGTGTCCGGCGA |
|  | dsGFP-R | taatacgactcactatagggCACCTTGATGCCGTTCTTCT |

Supplementary Table 2. The species of 32 differential lipid metabolites

| **Index** | **Compounds** | **Class I** | **Class II** | **VIP** | **Type** |
| --- | --- | --- | --- | --- | --- |
| LIPID-N-0003 | isolithocholicacid | ST | BA | 1.789 | up |
| LIPID-N-0006 | Ursodeoxycholicacid | ST | BA | 1.701 | up |
| LIPID-P-1498 | Cer(d18:0/16:0(2OH)) | SP | Cer-ADS | 1.928 | up |
| LIPID-P-1513 | Cer(t18:0/24:0(2OH)) | SP | Cer-AP | 2.055 | up |
| LIPID-P-2621 | Cer(d28:2/31:1(2OH)) | SP | Cer-AS | 1.873 | up |
| LIPID-P-2447 | Cer(t18:0/24:0) | SP | Cer-NP | 2.186 | up |
| LIPID-P-0121 | Cer(d18:1/18:1) | SP | Cer-NS | 1.826 | up |
| LIPID-P-0111 | Cer(d18:1/18:0) | SP | Cer-NS | 2.141 | up |
| LIPID-P-0109 | Cer(d18:1/16:0) | SP | Cer-NS | 1.859 | up |
| LIPID-P-0124 | Cer(d18:1/24:1) | SP | Cer-NS | 1.927 | up |
| LIPID-P-0134 | Cer(d18:2/16:0) | SP | Cer-NS | 2.309 | down |
| LIPID-P-2072 | Cer(d18:1/18:2) | SP | Cer-NS | 1.570 | up |
| LIPID-N-0609 | CerP(d18:1/18:1) | SP | CerP | 1.954 | down |
| LIPID-P-1834 | DGDG(18:4_18:4) | GL | DGDG | 2.562 | down |
| LIPID-P-1830 | DGDG(18:3_18:4) | GL | DGDG | 2.386 | down |
| LIPID-N-0030 | (±)18-HEPE | FA | Eicosanoid | 2.669 | down |
| LIPID-N-0099 | FFA(22:4) | FA | FFA | 1.773 | up |
| LIPID-N-0094 | FFA(18:3) | FA | FFA | 1.796 | up |
| LIPID-N-0092 | FFA(22:2) | FA | FFA | 1.881 | up |
| LIPID-N-0091 | FFA(20:2) | FA | FFA | 1.839 | up |
| LIPID-N-0086 | FFA(20:1) | FA | FFA | 1.805 | up |
| LIPID-N-0102 | FFA(22:5) | FA | FFA | 1.797 | up |
| LIPID-P-0305 | HexCer(d18:1/22:1) | SP | HexCer-NS | 1.776 | up |
| LIPID-P-0306 | HexCer(d18:1/24:1) | SP | HexCer-NS | 1.804 | up |
| LIPID-P-0394 | LPC(O-16:0) | GP | LPC-O | 2.514 | down |
| LIPID-P-0438 | LPE(P-16:0) | GP | LPE-P | 2.441 | down |
| LIPID-N-0665 | LPG(20:2) | GP | LPG | 2.350 | down |
| LIPID-P-0560 | PC(O-18:1_20:4) | GP | PC-O | 2.409 | down |
| LIPID-P-0555 | PC(O-18:0_20:4) | GP | PC-O | 2.453 | down |
| LIPID-P-0554 | PC(O-16:0_20:4) | GP | PC-O | 2.394 | down |
| LIPID-P-0652 | PE(P-16:0_20:4) | GP | PE-P | 1.813 | down |
| LIPID-N-1427 | PI(15:1_18:3) | GP | PI | 2.585 | down |
